# GPs’ acceptability and feasibility for using point-of-care tests for cancer in primary care: a qualitative interview study

**DOI:** 10.3399/BJGPO.2024.0191

**Published:** 2025-08-27

**Authors:** Anam A Ayaz-Shah, Richard D Neal, Kelly E Lloyd, Matthew J Thompson, Samuel G Smith

**Affiliations:** 1 Leeds Institute of Health Sciences, University of Leeds, Leeds, UK; 2 Exeter Medical School, University of Exeter, Exeter, UK; 3 Department of Family Medicine, University of Washington, Seattle, WA, USA

**Keywords:** qualitative research, diagnosis, cancer, primary health care, point-of-care testing, near-patient testing

## Abstract

**Background:**

Primary care is the first point of contact for patients with symptoms suspicious of cancer. The availability of reliable, rapid diagnostic cancer tests, at the ‘point of care’, have the potential to expedite diagnosis, and support timely management of patients.

**Aim:**

To explore the acceptability and feasibility of using point-of-care tests (POCTs) for detecting cancer among UK GPs, including barriers and facilitators to uptake.

**Design & setting:**

A qualitative semi-structured interview study with 32 UK GPs.

**Method:**

Online and telephone interviews guided by the theoretical framework of acceptability were conducted. The data were analysed inductively using framework analysis.

**Results:**

GPs found POCTs acceptable if they were accurate, well-designed, and supported by robust evidence. Funding for tests and implementation resources were crucial, with an expectation of remuneration for their time. GPs believed POCTs could improve patient triage, reduce secondary care referrals, and facilitate clearer communication of referral decisions with patients. Concerns included potential workload increase, and overtesting in patients. Facilitators for uptake included recommendations in guidelines, peer acceptance, and comprehensive training. However, low awareness of POCTs among GPs and slow innovation adoption within the NHS were considerable barriers.

**Conclusion:**

Most GPs welcome the use of POCTs for cancer detection in primary care; however, this will require substantial system-level changes. We highlight the relevant considerations and challenges that need to be addressed before uptake. This study also calls attention to wider innovation implementation issues that should be considered by GPs, test developers, policymakers, and stakeholders.

## How this fits in

The availability of cancer point-of-care testing in primary care has the potential to expedite diagnosis, reduce diagnostic oversight, and improve triage of patients to secondary care. Despite their availability, cancer point-of-care tests (POCTs) are not currently used in general practice and research assessing their utility is limited. This is the first study to explore the acceptability and perceived feasibility of cancer POCTs among GPs, highlighting the facilitators and challenges to uptake. GPs believed there is value in the use of good-quality cancer POCTs, subject to financial and resource considerations.

## Introduction

Cancer remains the leading cause of death in many countries, and more than half of these deaths are preventable.^1^ The significance of earlier diagnosis for improving patient outcomes is widely accepted.^2–4^ This has led to a myriad of screening and diagnostic innovation, including multi-cancer detection tests,^5^ imaging breakthroughs such as colon capsule endoscopies,^6^ and AI-driven clinical decision support tools.^7^ There is also an increased interest in system redesign by developing quick diagnosis pathways, outside of hospital-based settings.

In the UK, primary care is usually the first point of contact for patients presenting with symptoms of possible cancer and plays an increasingly important role in early detection. Patients presenting to their GPs with alarm symptoms are usually referred to a specialist on the urgent cancer pathway, following one or two consultations.^8^ For patients with cancers that are rare or have less distinctive characteristics, this can take three or more GP consultations,^9^ and represents missed opportunities for diagnosis.

About 90% of patients referred through urgent suspected cancer pathways are not diagnosed with cancer.^10^ This has implications for healthcare system capacity and patient experience, as many patients have symptoms requiring further assessment, which the current two-week wait (2WW) pathway does not address.^11^ In non-cancer settings, it has been suggested that point-of-care tests (POCTs) could improve triage and reduce unnecessary secondary care referrals.^12,13^

POCTs are hand-held clinical tests that can be performed during a consultation and usually provide results within minutes. Examples of existing POCTs include the UBC^®^ Rapid test for bladder cancer, CancerCheck^®^ PSA and PSAwatch for prostate-specific antigen measurement.^14^ An emerging innovation is the Breath Biopsy^®^, designed to detect lung cancer via volatile organic compounds.^15^

Point-of-care testing is a dynamic, multibillion-dollar industry.^16^ Several POCTs for cancer are commercially available or currently under development.^17^ Despite their availability, uptake of cancer POCTs in UK general practice has not occurred. To some extent, limited robust research evaluating the clinical need, efficacy, and utility of rapid tests may explain this gap between innovation and adoption. This is consistent with research that found, despite the growing market for and development of POCTs, evaluation studies mainly focused on the technical performance of tests and did not consider factors that were important to clinicians.^18^ As key stakeholders in primary care, it is important to assess the acceptability and perceived feasibility among GPs.

Acceptability has been defined as a multi-faceted concept reflecting how individuals involved in a healthcare intervention perceive its appropriateness, based on anticipated or experienced cognitive and emotional responses.^19^ While feasibility is less clearly defined in the literature, in this study, perceived feasibility refers to the extent to which clinicians believe POCTs could be integrated into practice.

This study aimed to explore and understand aspects of GP acceptability and perceived feasibility of using POCTs for detecting cancer in primary care, including perceived facilitators and barriers to adoption.

## Method

This study was reported in accordance with standards for reporting qualitative research.^20^ The study information was advertised via a market research company (www.dynata.com) and 53 participants expressed interest in the study. Of these participants, 47 were invited via email based on the order they signed up to participate, and 32 participants took part in the study. Participants were invited until the research team agreed saturation was reached. GPs practising in the UK were included in the study. Semi-structured interviews were conducted, either online (Microsoft Teams) or via telephone. Interviews were carried out by the lead author who had training and experience in conducting qualitative interviews. Consent forms and demographic information were completed online before participation.

The theoretical framework of acceptability (TFA)^19^ is a widely cited model for studying acceptability, comprising seven constructs that assess various aspects of acceptability alongside an overarching global acceptability construct. Our topic guide included one question for each construct and an additional question for overall acceptability (Supplementary Table 1). Questions addressing perceived feasibility were developed by the lead author in collaboration with the research team (Supplementary Table 2). Further probes and follow-up questions were incorporated iteratively in the early stages of data collection. Interview questions were framed around POCTs for cancer broadly rather than using specific exemplar tests. All interviews were professionally transcribed verbatim with identifiable information removed and replaced with pseudonyms.

Data analysis was guided by the framework analysis approach,^21^ managed on NVivo software (version 14). Initially, two authors (AAS and KEL) independently reviewed and inductively coded the same three transcripts. Both authors had previous experience with conducting inductive thematic analysis. Inductively coding the transcripts ensured themes not covered by the framework were identified, as previously suggested in the literature.^22^ The researchers discussed each coded selection and their interpretations of the data. Based on these discussions, AAS developed the analytical framework, grouping codes into categories. A further two transcripts were independently indexed using the analytical framework by AAS and KEL, which was refined in line with the emergence of new codes. This version was reviewed and discussed within the wider research team. The framework was used to index all remaining transcripts by AAS in NVivo. This process was done iteratively, with any new emergent codes or refinements to the framework discussed within the team.

## Results

Interviews lasted between 24 and 49 minutes. The final framework consisted of 51 codes, grouped into 13 categories (Supplementary Box S1). The data were charted into a framework matrix, and themes were generated from the dataset. Thirty-two GPs were interviewed between July and September 2023. Participant characteristics are presented in Table 1. Just over half (59%, *n* = 19) of the GPs were male and most were from England (88%, *n* = 28). GP partners made up the largest proportion of the sample (53%, *n* = 17) and just over one-third (34%, *n* = 11) reported having extended roles. Most GPs had been doctors for more than 20 years (47%, *n* = 15).

**Table 1.** Participant characteristics

Participant characteristics	*n* = **32**
**Sex**	
Male	19 (59%)
Female	13 (41%)
**Country of practice**	
England	28 (88%)
Scotland	3 (9%)
Wales	1 (3%)
**GP status**	
Partner	17 (53%)
Salaried or locum	14 (44%)
GP retainer	1 (3%)
**Years as a doctor**	
6-9	1 (3%)
10–14	5 (16%)
15–20	11 (34%)
>20	15 (47%)
**Specialist training**	
GPs with extended roles	11 (34%)

There was an overlap between constructs of acceptability and perceived feasibility, as POCTs were usually acceptable to GPs when adoption was considered feasible. Most GPs found the potential use of POCTs for cancer in primary care acceptable and feasible, subject to certain proviso. The key themes identified were as follows: (1) caveats to GP acceptability for POCTs; (2) the utility of POCTs in primary care; and (3) facilitators and barriers to POCT uptake. These are explored in more detail below.

### Theme 1: Caveats to GP acceptability for POCTs

#### Evidence for accurate, well-designed tests is crucial

Diagnostic certainty was the most important component for acceptability, emphasised by all GPs. Participants felt the usefulness of a cancer POCT would primarily depend on its sensitivity, specificity, and reliability in detecting cancer in primary care populations. It was believed that an accurate test would be beneficial, bring efficiency to cancer investigations, and be welcomed by the medical community:

*‘… if it’s accurate and useful, then I can imagine it would be a win-win for primary and secondary care if the commissioners get together and develop fast-track pathways for these tests.’* (GP23)

GPs acknowledged most tests are not 100% accurate, but still expected tests to demonstrate high sensitivity and specificity, with low rates of false positives and false negatives. Many participants suggested that sensitivity and/or specificity should be nearly 100%:

*‘… if we had a test that was 100% sensitivity and the test came back negative, then you would be telling the patient, … if you have it, then … this test would definitely have picked* [it] *up … the sensitivity,* [of] *even the gold standard, for example, colonoscopy … that will still miss some cases, nothing is 100% perfect. So we’d have to make sure that it is a decent test.’* (GP18)

They felt having too many false positives or negatives with a cancer test could have serious consequences for early cancer detection:

*‘... if there’s a high rate of false positives, it could cause unnecessary anxiety, unnecessary investigations … if there’s a high rate of false negatives and it’s not picking up enough then cases could be missed, and we could’ve been using other tests to detect it earlier more efficiently.’* (GP4)

Point-of-care COVID-19 and PSA blood tests were frequently cited as examples of tests with problematic accuracy. Some participants emphasised that cancer POCTs should be at least as reliable as existing diagnostic tests to minimise the risks associated with false positives and false negatives:

*‘…* [if] *the sensitivity, specificity are comparable to the blood test that we do at the moment, then … I don’t see any major disadvantages, depending on what test it is of course.’* (GP31)

Participants also discussed the importance of having research-based evidence to support test efficacy before use. Some GPs emphasised the importance of having POCT research that was representative and generalisable to UK primary care populations:

*‘I think you would need to see evidence that it’s consistent with the patient group we’d be using it in first and foremost, so in my population I’m thinking about you know, a fairly undifferentiated population in an area of deprivation with … multi-ethnic population ...’* (GP7)

In addition, most GPs required cancer POCTs to be well-designed and easy to use. This included factors such as an easy to retrieve sample type (for example, finger-prick blood versus urine), quick turnaround of results, and straightforward equipment. Some GPs discussed the importance of having easy-to-read test results, which were preferably dichotomous and not subject to interpretation:

*‘It has to be user-friendly, especially when it comes to ... time demand. It has to be objective ... for example, the urine dipstick is a very unreliable test ... especially for cancer detection, you do need something that can be only read in one way, and it won’t be subject to interpretation ...’* (GP36)

This was particularly important when considering the possible role of other clinical staff when using POCTs:

*‘Patients aren’t necessarily only seeing GPs now, they’re often seeing ANPs* [Advanced Nurse Practitioners] *who are working very closely alongside us so ... it’s got to be easy enough ...’* (GP40)

#### Testing must be adequately resourced in all aspects of implementation

Many GPs stressed the importance of appropriate resources to ensure adoption of cancer POCTs would not impede or introduce delays to existing general practice workflows. External funding for the purchase and maintenance of testing equipment was expected, alongside resources to employ additional staff to support POCT uptake:

*‘ ... it would need to be financed and I think you would need additional personnel to be funded to perform the test because of the potential time implications to do the test.’* (GP30)

Several participants expressed concerns regarding an increased workload for already time-strained clinicians, and whether this would be fairly remunerated. This was mentioned particularly in the context of the shift in workload and responsibility from secondary care to general practice:

’ *... if we’re taking work away ... from hospital medicine, which is great, freeing up their appointments and so on, will that be fairly kind of recompensed in primary care, where we’re already overworked and overburdened?’* (GP13)

Additionally, some GPs suggested monetary incentivising of testing would play an important role in its acceptance by clinicians. One participant compared POCTs with glucose testing incentivisation:

*‘... if there is incentive involved, for example, I’m doing all our glucose tolerance tests in my practice because I get incentive, but 90% of practices are not doing it because they don’t get their*[s] *... for cancer also, probably you have to give some incentive and some practices will do it.’* (GP15)

GPs felt, contrary to popular belief, general practice was capable of incorporating an increased workload that had clear patient benefits, if it was adequately resourced:

*‘People just have this assumption that doctors are too busy, they can’t take any more work on, I think the problem is they can’t take unresourced work on. If there’s specific workload that’s thought to be of benefit to patients and there’s a resource behind it, then GP practices can recruit to those roles that can get that work done.’* (GP2)

### Theme 2: The utility of POCTs in primary care

#### POCTs could be a useful tool to support GPs in cancer detection

Most participants believed having access to POCTs could support them to make appropriate referrals sooner. GPs felt using POCTs would give them more confidence in their diagnosis, and decision making regarding possible cancer. This was discussed particularly in the context of patients presenting with vague symptoms, or when clinical suspicion was high, but symptoms did not qualify for 2WW pathways:

*‘I think it could help GPs to feel more confident in their referrals and it might help to kind of stratify patients who need to be seen really very urgently and those that still need to be seen but could potentially be seen with a little bit of a longer wait potentially.’* (GP16)

Participants also felt that doing a POCT would make it easier for GPs to communicate their diagnostic decisions with their patients, especially when patient–practitioner trust is lacking:

*‘ … if we can reassure* [the patient] *no, your symptoms are not cancer ... I suppose, it’s just the reassurance ... where patients might not be particularly believing of what the GP is telling them because they don’t have that trust in us, because everybody knows Dr Google’s better, if you’ve got a test that you can, again, objectively show them ...’* (GP18)

#### GPs’ perspectives on benefits and harms for patients and wider system

POCTs were thought to have several benefits for patients, and patient welfare and acceptability for testing were important to GPs. The advantages of patients getting an earlier diagnosis and referral were highlighted by many participants. It was felt that administering POCTs had the potential to reduce anxiety, reassure patients, and avoid unnecessary invasive tests:

*‘ … it brings tremendous benefits to patients potentially as well as clinicians by giving immediacy to results and reducing the anxiety and lag time to get results from tests that are done in other ways. It can reduce the number of invasive tests that need to be done and replace them with relatively non-invasive tests to rule-out patients that are low risk.’* (GP2)

Some participants felt rapid results would give patients no time to process a positive test result and questioned if primary care was equipped to communicate a positive diagnosis to patients. Additional concerns included a public and/or clinician demand driven increase in testing, and its implications for patients and the wider healthcare system:

*‘ So, if people are aware that this test is available you might get a deluge of patients who are probably inappropriate for the test who are requesting the test ...’* (GP23)

*‘... there’s considerable waiting times already in secondary care ... if we’re going to be doing these additional testing and potentially referring more patients then there obviously needs to be some system in place to make sure that actually these patients are then seen promptly.’* (GP29)

### Theme 3: Facilitators and barriers to POCT uptake

The inclusion of POCTs in guidelines and recommendations, such as those of the National Institute for Health and Care Excellence (NICE), were considered an important motivation for clinicians to incorporate their use in clinical practice. Clear recommendations on robust clinical pathways, and training around POCT use was thought to encourage uptake. GPs felt they would be more willing to use POCTs if they were being widely adopted by colleagues across general practice. The faecal immunochemical test (FIT) was often used as an example for this:

*‘ ... I’d probably want it to be in the guidelines that it’s recommended that we use it, whether that’s local guidelines or NICE guidelines or whatever ... if they become more well-known and particularly if they become part of guidelines, then that would definitely get GPs doing it. I know gastroenterology, colorectal two-week wait referrals, a lot of places now you have to do a FIT test first and there was a lot of pushback against that initially.’* (GP16)

It was also believed that the receptiveness of POCTs would be influenced by the communication and knowledge surrounding their introduction:

*‘… if you’re going to get a project done, you need some kind of champion and knowledge vendor and then the language that you use to different groups varies quite tremendously. The narrative is important as much as anything.’* (GP1)

Many participants admitted to being unaware of POCTs for cancer and felt this lack of knowledge was a contributory factor to the lack of uptake in general practice:

*‘So, yeah one of the things that attracted me about the study was, I know little about point-of-care testing for cancer, and I’ve certainly not encountered … it really at all. So, I think there’s the, it’s not been publicised and there’s little awareness of it among GPs say, as a group.’* (GP21)

One GP highlighted issues around delayed adoption of innovation within the NHS and the lack of centralised roll-out of interventions with recurrent funding as a considerable barrier:

*‘What you see at the moment is little, tiny pockets of enthusiasts that do good stuff, dissemination of innovative practice throughout the NHS is pretty slow, having hit my head against brick walls quite a few times … things like if it’s rolled out in small pilots without central thought, is there recurrent funding …’* (GP1)

## Discussion

### Summary

GPs broadly deemed POCTs for cancer in primary care acceptable and feasible provided they were accurate, tailored for use in general practice, and externally backed with resources needed for implementation. Concerns relating to feasibility mostly pertained to the potential increased workload for time-constrained clinicians and the funding aspects of test adoption. Other considerations included the wider impact of using POCTs for patients, on the health infrastructure, and diagnostic pathways. This was particularly noted in the context of whether patients would be managed promptly in secondary care following referrals.

Rapid testing is typically used in emergency settings where prompt clinical decision making is lifesaving, or in resource-limited settings where access to diagnostic testing is otherwise restricted. We speculated that clinicians might not be convinced that ‘same-day results’ for cancer are necessary, as a short delay would be unlikely to change prognosis. However, our interviews revealed, in addition to the fast turnaround time to results, GPs identified many other perceived benefits of using POCTs for cancer, such as improved triage and more confidence in their diagnosis, especially in patients who did not qualify for urgent suspected pathways. It was noted that cancer POCTs had a potential to reduce unnecessary secondary care referrals and help stratify patients with vague symptoms.

Strong research evaluating the clinical performance of POCTs in primary care populations, alongside evidence for patient acceptability and improved outcomes, were motivators for uptake. Recommendations from NICE or local guidelines were considered important facilitators for adoption. Interestingly, some GPs reported feeling more comfortable with using POCTs if they were widely accepted among colleagues, demonstrating the function of peer influence in this context. Participants often cited the FIT test roll-out as an example of an initially resisted intervention that became more acceptable with wider use.

The narrative and language used when introducing POCTs to clinicians was thought to be important, as was providing the right knowledge and training. Awareness of cancer POCTs was generally low among participants and a potential barrier to uptake, while delayed innovation adoption in the NHS and lack of centralised roll-out with sustained funding were considered a hindrance for widespread implementation.

### Strengths and limitations

The semi-structured interview design allowed us to explore aspects of acceptability based on an existing theoretical framework,^19^ while delving deeper into key issues critical for implementation from GPs’ perspectives. The use of the TFA for developing our topic guide ensured a comprehensive assessment of various and nuanced aspects of acceptability by prompting discussion on specific dimensions such as GP confidence in ability to use POCTs.

We adopted a flexible and iterative approach towards conducting interviews. This ensured that participants were guided through structured questions, but were encouraged to lead the conversation on issues most important to them, allowing us to capture aspects we had not previously considered. Additionally, this study provides insights for developing other cancer interventions in general practice.

Interview questions for the study were based on point-of-care testing for cancer broadly rather than using a specific POCT as an exemplar. This method effectively elicited GPs’ perspectives on the ideal characteristics of a cancer POCT, capturing evaluation and implementation issues more widely. However, clinicians’ limited knowledge about POCTs for cancer might have led to assumptions about a hypothetical test, resulting in less specific feedback.

Most study participants were experienced clinicians, with more than 15 years of practising as doctors. Therefore, our findings may not be representative of the views of junior clinicians in the workforce. GPs were predominantly from England, which could reflect a skew in perspectives more applicable to NHS England. This may be particularly important when considering differences in cancer pathway models and funding paradigms for tests between the three nations that participants were from (England, Scotland, and Wales; no responders were based in Northern Ireland).

This study focused on GPs’ perspectives, excluding other primary care clinicians. As GPs emphasised the importance of involving other clinical staff in POCT delivery, including their input may have provided alternative views on acceptability and perceived feasibility of POCTs in primary care.

### Comparison with existing literature

Echoing findings from previous studies assessing the general use of non-cancer POCTs in primary care,^23–27^ having well-designed tests that provide diagnostic certainty, emerged as important factors for GPs when considering the adoption in their clinical practice. Compared with these studies,^23–27^ accuracy was prioritised more by GPs in our research owing to the consequences of a misdiagnosis. False-negative test results can provide false reassurance, delay cancer detection and treatment, significantly worsening patient prognosis. Conversely, a false-positive result can cause undue anxiety and lead to unnecessary invasive testing. No test achieves perfect sensitivity or specificity; however, diagnostic uncertainty, interobserver variability, and the potential for errors owing to interpretation can be considerable concerns.^28^ GPs acknowledged this, advocating for dichotomous or numerical values for cancer POCTs to reduce uncertainty. Previously, the World Health Organization published the ASSURED criteria for the ideal POCT for use in infectious tropical diseases and sexually transmitted infections, with a particular focus on low-income countries.^29^ This included prerequisites regarding affordability, accuracy, and user-friendliness. These features aligned with the aspects GPs in our study identified as important for a cancer POCT.

In alignment with another study on POCTs for lower respiratory tract infections, we found GPs reported similar advantages for cancer POCTs.^30^ These included improved communication with patients, confidence in decision making, and better triaging of patients. Similar to previous research, system-level issues for implementation, such as time constraints in primary care, funding, and reimbursement models, were important aspects for consideration.^23–26^ However, owing to the qualitative design of our study, we cannot determine the prioritisation order of these variables, and this may vary depending on disease context.

Many GPs believed additional staff resources could mitigate the increased workload in response to POCT uptake. This contrasts with literature suggesting that employing non-GP clinicians does not necessarily reduce GP workload.^31^ The extent to which GPs envisioned other clinical staff supporting the administration of POCTs for cancer was not clear, and some may have implicitly anticipated hiring more GPs. Further enquiry is essential for assessing how additional staff would support the roll-out of POCTs for cancer.

Inclusion of POCTs in clinical guidelines was a facilitator for GP uptake. This was interesting because research indicates GPs sometimes find it challenging to adhere to guidelines^32^ and lack awareness of newer recommendations.^33^ GPs may instinctively prioritise clinical judgement over guidelines, which is justifiable given the important predictive value of GPs’ ’gut feelings’ in cancer diagnosis.^34^ Future efforts should support GPs in adhering to clinical guidelines and integrate the value of clinical acumen with evidence-based practice.

### Implications for practice

This study establishes the basis for introducing point-of-care cancer diagnostics in primary care by identifying GPs’ requirements and how they might be integrated. The NHS Long Term Plan aims to diagnose three in four cancers at an earlier stage by 2028. Achieving this goal will require a multifaceted approach, including optimising diagnostic pathways and integrating innovations to enhance GPs’ precision for cancer referrals. Over the past decade, urgent cancer referrals have nearly doubled,^35^ significantly burdening diagnostic services. However, in England only 7% of these investigations resulted in a cancer diagnosis,^35^ highlighting the potential for POCTs to improve triage and reduce referrals on urgent suspected cancer pathways.

Our findings indicated that integrating POCTs in primary care would require considerable system reform, which will need collaboration between test developers, clinicians, policymakers, and commissioners. Future evaluation studies must assess and report on aspects important to primary care clinicians such as patient benefit, risk, acceptability, and wider system feasibility, including cost-benefit analyses.

This study identified multiple factors that could impact GPs’ motivation to utilise POCTs. First, while adherence to clinical guidelines is important, clinical judgement often takes precedence. GPs are less likely to use a test if they are not convinced of its evidence, particularly regarding patient benefits and outcomes. Additionally, the introduction of a test relies on a combination of guideline recommendations, evidence, and peer opinions, demonstrating the influence of social factors.

Many GPs lack awareness and knowledge of cancer POCTs, which could hinder their adoption. This underscores the importance of providing adequate training and education if these tests are to be implemented, ensuring clinicians are confident in their use. The broader issue of slow translation and adoption of emerging innovations within the NHS was highlighted in this study. To keep pace with evolving cancer diagnostic technologies and fully leverage their advantages, it is crucial to reconsider and expedite the processes for integrating modern technologies into practice.

In conclusion, UK GPs felt POCTs can offer major benefits for cancer detection in primary care for clinicians, patients, and the wider healthcare system. However, important challenges, such as test accuracy and tailoring for general practice, must be overcome to realise these merits. Access to adequate financial and staff resources would be paramount for implementation, with careful consideration for managing the potential increased workload for clinicians. Addressing these challenges will help improve pathways to better detection and encourage the development of innovations such as multi-cancer POCTs that may be implemented in the future.

## Data

The dataset relied on in this article is available from the corresponding author on reasonable request.
